# Targeting autonomic nervous system as a biomarker of well-ageing in the prevention of stroke

**DOI:** 10.3389/fnagi.2022.969352

**Published:** 2022-09-15

**Authors:** Jean-Claude Barthelemy, Vincent Pichot, David Hupin, Mathieu Berger, Sébastien Celle, Lytissia Mouhli, Magnus Bäck, Jean-René Lacour, Frederic Roche

**Affiliations:** ^1^Physical Exercise and Clinical Physiology Department, CHU Nord, Saint-Étienne, France; ^2^INSERM U1059 Santé Ingénierie Biologie, Université Jean Monnet, Saint-Étienne, France; ^3^Section of Translational Cardiology, Department of Medicine, Solna, Karolinska Institutet, Stockholm, Sweden; ^4^Centre d’Investigation et de Recherche sur le Sommeil, Centre Hospitalier Universitaire Vaudois, Lausanne, Switzerland; ^5^Département de Neurologie, Hôpital Universitaire Nord, Saint-Étienne, France; ^6^Department of Cardiology, Karolinska University Hospital, Stockholm, Sweden; ^7^Laboratoire de Physiologie, Faculté de Médecine Lyon-Sud, Oullins, France

**Keywords:** aging, longevity, parasympathetic activity, stroke, ANS activity, epidemiology, neuroendothelial disease, inflammation

## Abstract

Stroke prediction is a key health issue for preventive medicine. Atrial fibrillation (AF) detection is well established and the importance of obstructive sleep apneas (OSA) has emerged in recent years. Although autonomic nervous system (ANS) appears strongly implicated in stroke occurrence, this factor is more rarely considered. However, the consequences of decreased parasympathetic activity explored in large cohort studies through measurement of ANS activity indicate that an ability to improve its activity level and equilibrium may prevent stroke. In support of these observations, a compensatory neurostimulation has already proved beneficial on endothelium function. The available data on stroke predictions from ANS is based on many long-term stroke cohorts. These data underline the need of repeated ANS evaluation for the general population, in a medical environment, and remotely by emerging telemedicine digital tools. This would help uncovering the reasons behind the ANS imbalance that would need to be medically adjusted to decrease the risk of stroke. This ANS unbalance help to draw attention on clinical or non-clinical evidence, disclosing the vascular risk, as ANS activity integrates the cumulated risk from many factors of which most are modifiable, such as metabolic inadaptation in diabetes and obesity, sleep ventilatory disorders, hypertension, inflammation, and lack of physical activity. Treating these factors may determine ANS recovery through the appropriate management of these conditions. Natural aging also decreases ANS activity. ANS recovery will decrease global circulating inflammation, which will reinforce endothelial function and thus protect the vessels and the associated organs. ANS is the whistle-blower of vascular risk and the actor of vascular health. Such as, ANS should be regularly checked to help draw attention on vascular risk and help follow the improvements in response to our interventions. While today prediction of stroke relies on classical cardiovascular risk factors, adding autonomic biomarkers as HRV parameters may significantly increase the prediction of stroke.

## Epidemiology of stroke and autonomic nervous system

The World Health Organization (WHO) describes stroke as a pandemic. From 1990 to 2010 the world burden of stroke increased significantly with regard to the recent increase in the numbers of patients, of deaths (20% increase), and of disability-adjusted life years (DALY; 16% increase) (Krishnamurthi et al., 2013). According to the WHO, the estimated incidence of stroke events in Europe is likely to increase from 1.1 million per year in 2000 to more than 1.5 million per year in 2025 in relation to demographic changes (Truelsen et al., 2006). Worldwide, the number of deaths due to stroke was 4.4 million in 1990 (Murray and Lopez, 1997). This prevalence is underestimated as many strokes do not exhibit clinical evidence and remain unnoticed; indeed, systematic autopsies of large populations disclosed a 12.9% pathological evidence of cerebral infarction, although any clinical sign of stroke had ever been experienced by these patients who died from other diseases (Shinkawa et al., 1995). Many studies also reported previous silent strokes in those patients investigated for acute stroke as in the Framingham (Kase et al., 1989) and the Copenhagen (Jorgensen et al., 1994) cohorts. In the Veterans Affairs Cooperative Study, 14.7% of cerebral scans showed previous silent brain infarctions (Hénon et al., 1995). This percentage reached 38.3% in the SEPIVAC survey (Ricci et al., 1993).

Death from stroke is not the only societal drama as 40% of survivors demonstrate severe sequelae, and 30% fall in depression in the year following a stroke. Dementia is a frequent evolution. In developed countries, each case from stroke occurrence to death costs almost $80,000 US or €70,000, with an annual amount of more than $100 billion US for the United States only (Feigin et al., 2014); The incidence may be largely under evaluated as there is approximately 15 silent stroke for one clinically patent stroke (Leary and Saver, 2003).

Autonomic nervous system activity and traditional risk factors are strongly related as ANS imbalance contributes to the creation of a pre-pathological milieu of composite risk factors, such as hypertension, diabetes or atrial fibrillation and alterations of endothelial homeostasis in favor of pro-thrombotic/proinflammatory state, eventually leading to increased risk of stroke (Saeed et al., 2005; Bäck et al., 2019; Carandina et al., 2021). Thus ANS monitoring is of importance in stroke prevention focusing on the imbalance characterized by a decrease in parasympathetic activity and a concurrent increase in sympathetic activity.

As ANS monitoring is most often based on mathematical analysis of successive ECG sinusal intervals (RR intervals), those which are neuro-controlled, subjects suffering from arrhythmias are often excluded from the studies. As a matter of fact, antiarrhythmic drugs alter significantly the HRV measurements, according to their class (Zuanetti et al., 1991).

## Autonomic nervous system and longevity

Autonomic nervous system parasympathetic outflow is a recognized predictor of longevity in centenarians, ANS decrease with aging being considered as a natural decrease of allostatic systems (Hernández-Vicente et al., 2020). In the general population, three longitudinal studies underlined the predictive value of decreased ANS activity for death, mainly cardiovascular, namely the ARIC (Atherosclerosis Risk in Communities), the ZUTPHEN and the FRAMINGHAM studies (Tsuji et al., 1996; Dekker et al., 1997, 2000). The same predictive value was attributed to a decrease in ANS after stroke of myocardial infarction (Kleiger et al., 1987). The ultracentenarians over 100 years of age have significantly higher parasympathetic activity than the elderly subjects from 81 to 100 years of age and the parasympathetic predominance may be the neuroautonomic feature that helps to protect ultra-centenarians against cardiovascular disease (Piccirillo et al., 1998). In animal models, increased sympathetic activity and decreased parasympathetic activity (Vanoli et al., 1991) are associated with a higher risk of sudden cardiac death (Kleiger et al., 1987; Bigger et al., 1993; Tsuji et al., 1996; Chen and Tan, 2007). Specifically, reduced 24-h HRV is independently associated with increased risk of myocardial infarction, congestive heart failure, death from cardiovascular disease, and total mortality (Kleiger et al., 1987; Bigger et al., 1993; Tsuji et al., 1996). Experimental evidence also suggests that the autonomic nervous system is implicated in the development of vascular atheroma and occlusion (Hamaad et al., 2004). On the other side, cholinergic stimulation protects endothelium by blocking endothelium cells activation and leukocyte recruitment during inflammation (Saeed et al., 2005). This opens a promising area for future research in the effects of common non-cardioactive drugs (Nicolini et al., 2012). In the cardiovascular health study (CHS), greater total leisure-time activity, walking distance, and walking pace were each associated with more favorable HRV indices, supporting cardiovascular benefits (Soares-Miranda et al., 2014).

## Autonomic nervous system, an easy tool

Stress is the response of the organism to the interoceptive and exteroceptive changes, i.e., to any biological, psychological, or environmental context change in daily life. The stress is most often beneficial but may become deleterious if solicited intensively or for a too long period of time. The regulation from ANS is immediate and even adapts to the perceived risk as well as to the perspective of future risks. This may even alter transgenerational epigenetical modulation of stress (Babenko et al., 2015).

Among the many adaptative changes, the stress mechanism activates the sympathetic nervous activity (ANS), with a mirrored decreased in parasympathetic nervous activity (PNS). This resultant prevailing sympathetic activity sets a multitude of negative side effects, particularly at the vascular level. There is also a biological cost to activate the biological regeneration through mechanisms including the sleep, vagal tone and tissue regeneration (Canini, 2019).

Stress response is traditionally considered to represent the *fly or fight* response to a significant external variation condition. Today, stress frequently deals with more common changes, such as hunger, thirst, temperature changes, walking, sleeping, speaking, eating, meeting people, listening noise or music, getting up or lying. All these actions require an adaptative answer of ANS to keep the biological equilibrium in spite of an unusual context. In that view, the stress response is present at many occasions and accompanies also many classical cardiovascular risk factors.

Several methods are available to measure ANS activity. Invasive methods include biological catecholamine measurements or direct nerve sympathetic activity measurement using microneurography (Wallin and Sundlöf, 1979). Although providing precise measures, these techniques do not measure parasympathetic activity and cannot be applied to large populations.

Fortunately, ANS activity can be also non-invasively investigated giving access to both the sympathetic and parasympathetic arms of ANS activity. The importance of heart rate behavior in response to exercise is illustrated by a predictive value for sudden death (Jouven et al., 2005). Even the notion of a relationship between longevity and heart rate as a simple measurement of ANS activity, has been raised (Jensen, 2019). Quantification of heart rate variability (HRV) provides more precise measurements than simple heart rate values. REF any change in ANS activity is reflected by HRV due to the very rich ANS innervation of the heart. In that view, the heart is an open window on the neurological regulatory ANS activity. The lack of variability of RR intervals means that there is no ANS activity to regulate heart rate, and often, no ANS activity at all. It means neuronal inactivity. This total lack of variability can be observed when a cerebral death has occurred in ICU, where heart rate becomes absolutely regular (Rapenne et al., 2000).

Analysis of heart rate changes allows quantifying ANS activity. Rapid vanishing changes in RR interval length are induced by the parasympathetic drive, due to the rapid elimination of its neurohormone, acetylcholine. These variations are called high frequency (HF) variations and thus represent the parasympathetic drive. Slower changes of RR intervals length are due to slower changes in catecholamine concentrations and are thus called low frequency (LF), they roughly represent the sympathetic drive which is while this LF frequency band also represents some parasympathetic activity (Brown et al., 1993; Lorenzi-Filho et al., 1999; Pichot et al., 1999).

Only the normal RR intervals are taken into account in HRV measurements, as their length is modulated by the autonomic nervous system, which is not the case for intervals linked to ectopic beats which are due to local reentry or local enhanced automaticity. The HRV evaluation implies first a reading of Holter recordings in order to label beats as normal (N), ventricular ectopic beats (V or VE), or supraventricular ectopic beats (S or SVE). This reading also gives access to the length of each normal to normal (NN) beat. HRV is then based on mathematical calculations on these consecutive NN intervals. Once the set of RR (NN) intervals is established, two classical mathematical approach to quantify the autonomic nervous system activity which modulate the NN length variations, namely the temporal and the frequential approaches (Task Force, 1996).

The temporal approach is based on simple standard deviations (SD) of these NN intervals, usually calculated on 24-h periods. The way the SD will be calculated will give several results. The first result is global autonomic activity calculating the SD of all RR (NN) intervals of the set, and it is called SDNN. Then, to take into account fast RR length variations from one RR interval from the previous, it is calculated the SD of the root mean square of the mean of the successive differences of consecutive RR (NN) intervals squared, giving a representation of the parasympathetic activity. It is called RMSSD. Referring to the sympathetic activity, the SD of RR (NN) intervals are first calculated on consecutive 5 min intervals, the mean of these SD is thus appropriate to reflect sympathetic HRV changes which are observed on longer periods than parasympathetic ones. It is called SDNNIDX. One last measurement which differs slightly from these SD and means, is the percentage of RR intervals differing for more than 50 ms from the previous one. It is a parasympathetic parameter. It is called PNN50.

The second set of mathematical approach is based on the analysis of cyclical variations of RR (NN) as a sinusoidal signal. This approach is most often performed through a Fourier Transform (FT), which identifies regular repetitive patterns that can be described as sinusoids. This is a general law that is applied to many periodic signals including physiological signals. It is a new mathematical space where signal modulations can be characterized by a frequency and amplitude modulation. Entering the Fourier space allows thus to quantify repetitive fast changes by fast sinusoids and repetitive slow changes by slow sinusoids. In RR length modulation physiology, the slow and fast changes are included in bounds calculated using pharmacological blocking of sympathetic and parasympathetic activities (Pomeranz et al., 1985). These bounds are 0.04 – 0.15 Hz for the low frequencies (LF) representing the sympathetic activity and 0.15 – 0.40 Hz for the high frequencies (HF) representing the parasympathetic activity. This gives also the possibility to calculate a ratio between the sympathetic activity and the parasympathetic activity, the ratio LF/HF, which represents the sympathetic predominance.

Indeed, while the representativity of parasympathetic activity by the HF band of Fourier transform is widely accepted, the representativity of sympathetic activity by the LF component is slightly more questionable since the LF band represents both parasympathetic and sympathetic activities, and does not correlate well with the MSNA activity, this latter being the reference value for sympathetic activity (Saul et al., 1990). The values should also be assessed against total spectral power (Ptot). HF and LF values are thus better assessed when compared to each other, and their ratio LF/HF is a good indicator of ANS imbalance (Pagani et al., 1984), when used in normalized units as well, although this last index remains questioned (Billman, 2013). There is also significant respiratory influences on HRV that affect not only the HF component, but could affect LF as well, depending on the breathing pattern (Brown et al., 1993; Lorenzi-Filho et al., 1999).

These temporal and frequential methods share a limitation in averaging ANS activity on the period analyzed, thus missing transitions in ANS modulation. A first answer is to analyze separately diurnal and nocturnal values. The nocturnal values are of particular interest as they are independent of most environmental stimuli and thus better represent the ability of ANS to activate the parasympathetic drive.

**FIGURE 1:**
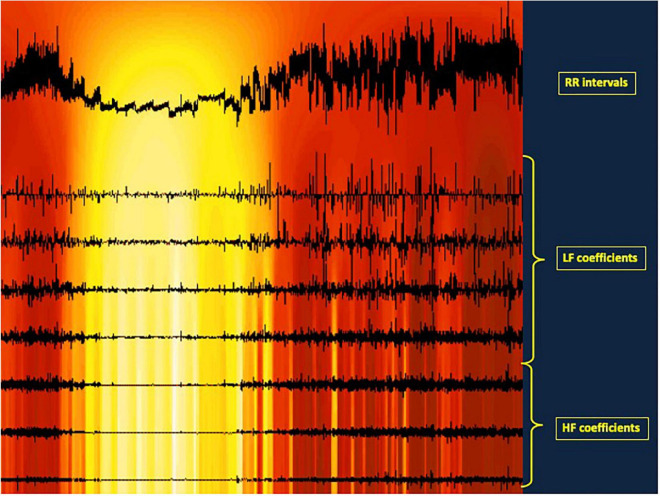
Illustration of a wavelet analysis for HRV. The upper trace represents the RR intervals length. The seven horirontal lines illustrates the autonomic nervous system activity, the three lowest lines representing he parasympathetic activity, the four upper lines the sympathetic activity. On each horizontal line, a vertical line is traced each time a parasympathetic or a sympathetic activity is detected, i.e., each time the RR length changes at a fast time (HF) or at a slower pace (LF). The additions of these coefficients give the total parasympathetic and sympathetic activity, respectively. In addition, in that experiment, a transient profound artificial aging was induced as a result of intravenous atropine administration at 20-min intervals. That administration shortens progressively th RR intervals. At the same time, the autonomic coefficients representing heart rate variability decrease then disappear. Then they reappear also progressively after the last atropine administration. This illustrates how a wavelet analysis of HRV can measure both the quatitative variations of parasympathetic and sympathetic activity by summing the coefficients along the period analyzed, but can also localize HRV change along the time. The colored background illustrates the ANS activity along the time from high activity in red to low activity in yellow.

It is also possible to pick up transitional values of ANS activity using a specific frequential measurement based on wavelets analysis (Pichot et al., 1999, 2016). This allows to go beyond the limitation of stationarity needed for temporal and frequential methods. Stationarity is rather difficult to obtain but this status benefits from a general tolerance. Wavelet analysis is of particular interest in experimental conditions such as drug assessment, autonomic answer to physical exercise, or stress evaluation under any environmental changes. The wavelet transform (WT) presents other advantages over the FT. First, the shape of the analyzing wavelet can be freely chosen, and thus is not limited to the sinusoid shape of the Fourier Transform, owing to more accurate measurements. Specific analyzing shapes of interest for EKG were published by Daubechies (1992). The second advantage of WT is the localization of the fitting between the analyzing shape and the analyzed signal, allowed by displacing the analyzing signal along the analyzed signal and thus identifying precisely the time where the change occurs (Figure 1).

Beyond these so-called linear approach of HRV, representing its complexity, non-linear approach gained a great interest (Acharya et al., 2004; Stein et al., 2005; Maestri et al., 2007). In fact, RR interval series demonstrating identical statistical linear properties (mean and SD) and power spectra can differ profoundly in terms of the “fine texture” of the rhythm (Nicolini et al., 2012).

These variables are the Poincaré plot (Kamen et al., 1996), the fractal analysis (Peng et al., 1995), the entropy, heart rate turbulence (Bauer et al., 2008), deceleration and acceleration capacities (Bauer et al., 2006), empirical mode decomposition (Balocchi et al., 2004), largest Lyapunov exponent (Wolf et al., 1985), symbolic dynamics (Porta et al., 2001), and empirical mode decomposition (Balocchi et al., 2004), among others. The readers interested may find some details in a dedicated paper from Pichot (Pichot et al., 2016). Unfortunately, these promising variables were seldom chosen due to the lack of available software before the publication of Pichot’s software, HRVanalysis (Pichot et al., 2016). Fortunately, we hope that past data may be reanalyzed using this software. New biomarkers gain a significant place as predictors of stroke as they did in postmyocardial infarction (Stein et al., 2005). Data about HRV in the elderly may be corrected to take into account ECG fragmentation, which may artificially increase variability in that population (Costa et al., 2017).

Spontaneous baroreflex (BRS) measurement is another non-invasive approach to ANS measurement. Smooth muscle in arteries, arterioles, and veins and pericytes in capillaries receive a rich autonomic innervation. The baroreflex measures the parasympathetic response to variations in blood pressure. The test is performed by analyzing the increase or decrease in blood pressure and the corresponding lengthening or shortening response of the following interval RR. This measurement is made possible without pharmaceutical administration due the spontaneous permanent change in systolic blood pressure (SBP) from one beat to the following. To do that, the ECG and blood pressure have to be measured simultaneously to belong to the same time frame. The non-invasive blood pressure is measured continuously non-invasively via fast digital balloon counter pressure (Peňáz, 1973). The interest in baroreflex measurement comes from its strong representation of parasympathetic activity as the measure checks at the same time the quality of the sensor of blood pressure located in the carotid body, and the ANS neuronal complete circuitry going from the sensor to the target, here from the carotid to the brainstem then to the heart (Seravalle et al., 2015). Arterial baroreceptors in the carotid sinuses and aortic arch sense pressure changes, and in response to an increase blood pressure they inhibit efferent sympathetic neurons leading to vasodilatation, and they influence the heart rate. They also decrease renin by limiting sympathetic renal outflow (Kaufmann et al., 2020).

Another approach is to evaluate blood pressure variability (BPV). As for HRV, high frequency and low frequency are very different. High frequency means fine adaptation, pulse to pulse, while low frequency means late adaptation trying to catch up the physiological target, but so late that an excessive adaptation is the rule. What is not continuously and finely adjusted will need later stronger corrections, hence the difference meaning of high frequency signing a permanent fine adaptation and low frequency signing a late adjustment.

Arterial stiffness is another approach of ANS activity. This is used on short term variations as a marker of sleep apnea by measuring the pulse transit time (Contal et al., 2013). On the long term, parameters of arterial stiffness, pulse pressure and its other components, are strong predictors of mortality and cardiovascular outcomes (Gavish et al., 2022). Arterial stiffness takes into account vascular aging and thus endothelium health which depends on ANS activity (Abboud, 2010).

Normal values for ANS measurements are scarce. Guidelines for measurement have been published, as well as some normal values (Shaffer and Ginsberg, 2017), of which nocturnal values are given from the Hypnolaus study (Berger et al., 2022). Various aspects of HRV were explored as short term values (Lee et al., 2018), values in women (Kesek et al., 2009), values in periodic leg movements (Sforza et al., 2005), in patients suffering OSA (Qin et al., 2021), and in those presenting with metabolic syndrome (Assoumou et al., 2012a).

Free software are today available, making possible calculation of HRV from almost any recording source with any available method (Pichot et al., 2016). This illustrates an emerging facilitation of the implication of ANS in cardiovascular risk assessment.

## Autonomic nervous system as a predictor for stroke occurrence

While today’s prediction of stroke risk relies on classical cardiovascular risk factors, adding autonomic biomarkers may significantly increase the prediction of stroke (Bodapati et al., 2017). The association of two HRV parameters significantly increased the predictive power from 0.61 for the CHS-score (Cardiovascular Health Study clinical stroke risk score, Stein et al., 2008) alone to up to 0.68 (*p* = 0.02). The implication of ANS unbalance in stroke occurrence in the general population is supported by the Framingham cohort. Specifically, a reduced 2-h HRV was independently associated with increased risk of myocardial infarction, congestive heart failure, death from cardiac and cerebral diseases, and total mortality (Kleiger et al., 1987; Bigger et al., 1993; Tsuji et al., 1996). Due to its short-term and long-term neurological interaction with vessels, ANS activity presents a strong relationship with cardiovascular diseases, including stroke. Since many risk factors are shared for MI and for stroke occurrences, the prediction brought through ANS imbalance predicts both vascular diseases as established in the Framingham cohort (Kleiger et al., 1987; Bigger et al., 1993; Tsuji et al., 1996). It should be emphasized that the term *cardiovascular* disease may be misleading as it focuses our attention on the heart disease while it downplays the *cerebrovascular* disease. Both terms should be associated to avoid this shadowing effect.

This makes of ANS imbalance a key factor of stroke occurrence. In The Framingham Heart Study, a 1 SD decrement in autonomic activity, as assessed by the biomarker HVR low-frequency power (LF, natural log transformed), was associated with 1.70 times greater hazard for all-cause mortality, including stroke. It is of note that the Holter recordings were obtained from 2-h diurnal recordings during routine examination in a subset of 1082 subjects (Tsuji et al., 1994). In a stepwise analysis including classical cardiovascular risk factors, SNA variable was the first to enter the model. The authors concluded that the estimation of HRV by ambulatory monitoring offers prognostic information beyond that provided by the evaluation of traditional risk factors (Tsuji et al., 1994). Thus HRV appears as a general cardiovascular (CV) risk marker, and that an individual has the age of his/her ANS activity (Tsuji et al., 1994). Recently, the Framingham Offspring cohort third addressed more specifically dementia and stroke prediction. Dementia was predicted by SDNN [HR (Hazard Ratio) per 1 SD, 0.61] and RMSSD (HR per 1 SD, 0.34). High resting heart rate was associated with increased stroke risk (HR per 10 bpm, 1.18).

Normal SDNN values were associated with lower stroke risk in men but not in women (HR per 1 SD, 0.46) (Weinstein et al., 2021). Nighttime HRV parameters are important as they are strong predictors of stroke in the Copenhagen Holter study, where eighty-one percent of the stroke occurred in the subjects with the lower half of nighttime SDNN (less than 38 ms; HR, 4.31) (Binici et al., 2011).

There are also epidemiologic evidences of ANS implication in clinically silent stroke which was found a predictor of patent stroke (AHA/ASA Scientific Statement). This can go from silent brain infarcts (SBI), white matter hyperintensities, or microbleeds (Smith et al., 2017), as established by the historical Hisayama study (Shinkawa et al., 1995). Age was a prominent factor, since in this cohort the percentage of subjects with silent infarcts increased with advancing age, from 4.4% in ages 40–49 to 19.3% in more than 80-year old humans. Other main established factors were hypertension, AF and diabetes mellitus. A recent meta-analysis including 14764 subjects underlined a 2.94 relative risk of clinically patent stroke following SBI, after adjustment for CV risk factors (Gupta et al., 2016).

Antiarrhythmic drugs alter significantly HRV, making HRV measurements different from basal values in these subjects (Zuanetti et al., 1991). However, this determines the exclusion of many subjects in the cohort studies as this may exclude the subjects with the most severe risk, determining some bias.

A study focusing on brain lacunae in elderly hypertensive patients underlined the lacunae to be rather related to ANS activity impairment than specifically to hypertension. As a matter of fact, while nocturnal dippers demonstrated an appropriate autonomic activity, extreme dippers, defined as having more than 20% nocturnal reduction of compared to their daily value, exhibited a markedly suppressed nervous activity during sleep. An extreme dip with a ratio night/day ≤0.8 was associated to lacunar events, and there was a correlation between the decrease of the HF nocturnal value as well as of the increase LF/HF ratio and the asleep/awake SBP ratio (Kario et al., 1997). In other words, an excessive sympathetic activity goes along with extreme nocturnal deep BP and brain lacunae. Non-dippers showed more advanced cerebrovascular disease than normal dippers, but less than extreme dippers. The depression of autonomic activity determining extreme dipping is also associated with brain lacunae (Kario et al., 1997). In a study comparing ANS through HRV, ambulatory blood pressure monitoring (ABPM), brachial artery endothelium-dependent flow-mediated dilation (FMD) and the intima-media thickness (IMT) of the carotid artery, it was shown an association between FMD and extreme dippers, reflecting an endothelial dysfunction and an increase in sympathetic activity (Hamada et al., 2008). Extreme dippers, with a night blood pressure decrease more than 20 percent of the diurnal values, are more prone to stroke, particularly to hemorrhagic stroke (Metoki et al., 2006). It is always important to consider that subclinical events of stroke on imaging and autopsy are much more frequent than clinically patent strokes, in the elevated proportion of 1–14 (Leary and Saver, 2003). Subclinical events are strong predictors of future clinical events (Gupta et al., 2016).

The way the ECG is recorded may influence the results, particularly if the recording covers less than a full nyctohemeral cycle. Indeed, from a day-time recording with only 2-h duration recording, as in the Framingham study, the data did not take into account the HRV nocturnal values, while they may be of great interest as the prevalence of nocturnal autonomic dysfunction is high in lacunar stroke patients even in the absence of the commonest sleep-related disorders. In this respect a full-day HRV ambulatory measurement may better describe the autonomic balance giving both day and night values (Hamada et al., 2008). As already stated, nighttime HRV parameters are strong predictors of stroke in the Copenhagen Holter study (Binici et al., 2011).

An abnormal HRV not only predicts a first-ever stroke but also contributes to increase the risk of stroke recurrence (Buratti et al., 2020). The prediction of stroke occurrence by low HRV parameters is also significant after a hip surgery (Ernst et al., 2021).

Whatever the recording duration, HRV remains a powerful approach for aging evaluation. Even HRV calculated on 12-s ECG recordings, as performed from standard 12-lead recording at bedside, made possible to establish the order in which people of the Zutphen cohort died within a 30-year time frame for any cause of death including cancer. Again, our age is that of our ANS activity (Dekker et al., 1997).

At the other end, in the Copenhagen Heart Study involving longer ECG recording durations up to 48 h, global HRV value represented by night-time SDNN global ANS activity was significantly associated with death prediction, even after additional adjustment for heart rate and other relevant biomarkers including serum triglycerides, hs-CRP, and NT-pro BNP. The hazard ratio reached 4.31, *p* 0.003, for those in the lower half of nighttime SDNN. In the same study, 24-h HRV variables were predictive of all-cause mortality, including stroke (Binici et al., 2011).

There is not one ANS parameter recognized as the best marker for stroke prediction. This comes from the lack of systematization of HRV measurements parameters, due to choices of duration of recordings, parameters analyzed, and the mathematical choice to assess the RR intervals dispersion. Global parameters as SDNN, are the most often introduced in the predictive algorithms since they are easier to understand and to perform. Within the choice of temporal measurements, RMSSD is also often chosen to assess the parasympathetic drive. Frequential analysis are also often used as they open the possibility to get the LF/HF ratio, which represents well the ANS imbalance. Diurnal recordings enhance the LF components as the daily life is much challenging than the nocturnal sleeping period which in turn better assesses the parasympathetic activity (Berger et al., 2022). Even the ANS evaluation in the Framingham study was conducted on short diurnal recordings. Free ANS software will benefit future, and possibly passed, studies in giving access to every ANS parameters (Pichot et al., 2015).

For many studies, a decreased sympathetic activity was the most prominent predictor of cardiovascular events. This can be explained as this is a worsening of ANS imbalance. Parasympathetic activity is not taken into account in the Framingham study as only diurnal measurement were performed, a time where the parasympathetic activity is spontaneously rather low and so variations in parasympathetic activity cannot be measured easily and if measured will be hardly significant (Tsuji et al., 1994, 1996). Conversely, an increase in parasympathetic activity, as obtained through vagal nerve stimulation (VNS), will decrease the sympathetic drive (Clancy et al., 2014).

Today, prediction of stroke brought through ANS measurement was mostly performed using SDNN, RMSSD, LF, and HF variables. This approach should be improved using more variables available to researchers including baroreflex which considers not only the ANS circuitry but also the quality of the carotid sensors in the baroreflex.

Ischemic stroke and hemorrhagic stroke are both concerned by ANS prediction. There are both related to vascular disease linked to classical risk factors, as hypertension, dyslipidemias, metabolic syndrome, inflammation, endothelial disease, and lack of physical activity, each being strongly associated to ANS imbalance (Jiang et al., 2022). As a matter of fact, prevention of both stroke types relies on the same guidelines (Caldwell et al., 2019).

Stress allows an efficient answer to aggressions, increasing the chances for survival, but an excessive intensity or duration is deleterious. ANS measurement may thus participate in the prediction of vascular events by identifying the excess of adrenergic activity, offering means of stroke prediction. However, no specific study yet describes predictive differences in ANS parameters for predicting ischemic stroke versus haemorrhagic stroke.

**FIGURE 2:**
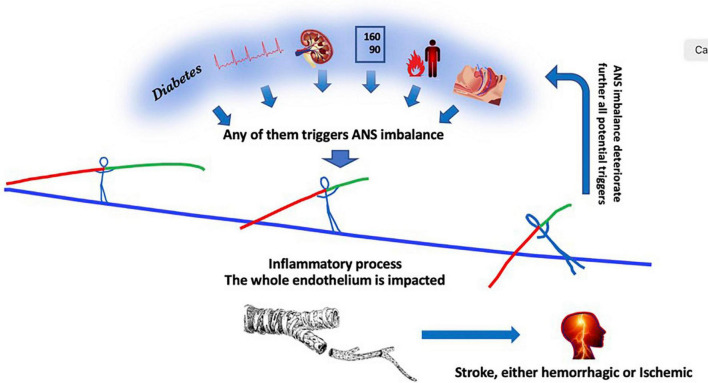
From left to right, diabetes, atrial fibrillation, chronic kidney disease, hypertension, inflammation, sleep apnea disorders, any of them contribute independantly to a global decrease in ANS activity with creates a predominance in the sympathetic activity of the remaining ANS activity. The autonomic nervous system activity imbalance determines in turn a global excess in circulating inflammatory products which in turn impacts the whole 300 square meter endothelium monolayer tissue, ending finally in a stroke. The ANS imbalance crushes further endothelium function which in turn increases multi-organ damages, aggravating the deleterious process.

In this context, there is nothing surprising that the diseases including the most severe altered ANS values are also those determining the shortest life duration and the more cerebrovascular complications. This is true in many chronic diseases and we will review some of them (Figure 2). These diseases often present multiple factors.

It is important to consider the role of autonomic system in stroke recurrence as it conveys the same predictive value than after a transient ischemic attack (TIA) (Guan et al., 2018). Measuring ANS in TIA allows to identify high risk sub-populations which may benefit from the warning (Guan et al., 2019). This is in concordance with the risk for a first stroke (Kleiger et al., 1987; Bigger et al., 1993; Tsuji et al., 1996).

### Autonomic nervous system as a predictive factor for stroke occurrence in the context of diabetes mellitus

The ANS circuits in diabetes are considered an integrative network centered on brainstem modified by and affecting diabetes-induced CVD (Espinoza and Boychuk, 2020). Even short periods of provoked hyperglycemia stimulate sympathetic activity with attenuation of parasympathetic activity (Majeed and Yar, 2020). At 60 years of age, the subjects of the PROOF study show a strong correlation between ANS dysregulation and their dysmetabolism. Furthermore, their ANS deactivation measured through baroreflex was proportional to the metabolic disorder (Assoumou et al., 2012a).

Autonomic nervous system activity is a stronger predictor of stroke in subjects with type 2 diabetes mellitus compared with the general population. Night-time HRV identified diabetics with cardiovascular events, including stroke; at an average follow-up of 14.4 years with a better fitting of the receiver operating curve (ROC) from 0.704 to 0.765 when adding SNA to conventional risk factors (Hadad et al., 2021). Progressive HRV worsening also predicted ischemic stroke independently of classical cardiovascular risk factors in diabetics (Yun et al., 2018). According to the authors, a one-unit increase of their combined predictor index increased by almost 10% the probability of events (Lai et al., 2021).

The ARIC cohort study also showed HRV to be a powerful predictor of cardiovascular sudden death during a mean follow-up of 13 years, with an increase of up to 27% in sudden death associated with low HRV (Fyfe-Johnson et al., 2016). While in their first publication the authors did not specifically analyze stroke occurrences, it is however well established that stroke is almost as frequent as coronary artery disease. A more recent publication relating a 22-year follow-up demonstrated a predictive HRV value for incident stroke, but only in the diabetic patients of the cohort (Fyfe-Johnson et al., 2016).

### Autonomic nervous system as a predictive factor for stroke occurrence in the context of chronic kidney disease

Chronic kidney disease (CKD) is associated with an increased risk of both ischemic and hemorrhagic strokes, in a range of 10–33 per 1000 patient-years depending on study population and design (El Husseini et al., 2014). In addition to the established high cardiovascular risk, the altered autonomic control in hemodialyzed patients further increases the probability of major adverse cardiac events and stroke (Kao et al., 2019). Lower HRV values, mainly low frequency, significantly predicts higher risk of cardiovascular disease (CVD) (Chandra et al., 2012). There is a relationship between the decline in glomerular filtration rate and autonomic neuropathy (Clyne et al., 2016). Renal failure increases sympathetic activity and overrides the stimulation by baroreceptor deactivation during hemodialysis (Boero et al., 2001).

Therapeutic targets for sympathetic activity decrease were proposed in CKD (Seravalle et al., 2021). Although the results of sympathetic renal nerve ablation were first very encouraging toward hypertension, with a profound decrease of systolic and diastolic blood pressure, there is no study analyzing long-term clinical improvement in inflammatory markers (Seravalle et al., 2021). A pilot study based on 1 month non-invasive VNS in CKD showed changes in tumor necrosis factor (TNF), interleukine-1 and -10 (IL-1 and IL-10) concentrations that did not reach statistical difference (Hilderman and Bruchfeld, 2020). Another VNS pilot study in hemodialysis patients determined a decrease in high-sensitivity C-Reactive protein (hsCRP) but not in TNF, IL-1b, or IL-10 (Hilderman and Bruchfeld, 2020). While this suggests that there may be a potential for cholinergic modulation in CKD patients, further studies are needed adding control of other conditions as sleep quality (Kadoya et al., 2021). However, in renal transplant recipients, a complete denervation of the native diseased kidneys by bilateral nephrectomy decreases cardiovascular risk (Obremska et al., 2016).

### Autonomic nervous system as a predictive factor for stroke occurrence in the context of inflammation

Chronic systemic inflammation, which is associated with parasympathetic inactivity (Tracey, 2002), is a well-recognized cardiovascular risk factor independently of hypercholesterolemia (Ridker et al., 1998, 2000). This is in line with contribution of chronic inflammation to the residual risk of myocardial infarction and stroke in the absence of elevated levels, or after attaining low levels of low-density lipoprotein cholesterol. The Framingham study, demonstrated ANS activity to hold a strong predictive power, higher than that of cholesterol (Ridker et al., 2002). Conversely, statins reduce sympathetic activity (Lewandowski et al., 2015).

The relationship between cardiovascular disease and inflammation evokes the notion of a role of low ANS activity as a possible inflammation-regulator extended to cardiovascular disease. This major marker of vascular aging should be of interest in primary prevention (Ridker et al., 1998). This is further reinforced by the increased cardiovascular risk and autonomic dysfunction in systemic chronic inflammatory diseases such as rheumatoid arthritis (Hupin et al., 2021), and obesity. Decreased parasympathetic activity is associated with central adiposity and higher SBP, indicative of increased metabolic risk, already at age 5–6 years (Vrijkotte et al., 2015). When a follow-up is performed, it can be seen that increased sympathetic activity predicts an increase in metabolic abnormalities and hypertension over time (Palatini et al., 2006; Licht et al., 2013). The PROOF (Prognostic Indicators of Cardiovascular and Cerebrovascular Events) study confirms a strong correlation between ANS dysregulation and inflammation in subjects aged 65 years old issued from the general population (Dauphinot et al., 2009, 2012; Assoumou et al., 2011). In this study, classical waist circumference clinical factor is strongly associated to inflammation (Assoumou et al., 2011) and increased inflammation predicts in its turn hypertension (Dauphinot et al., 2009).

In a study including people from the Women’s Health Study, adding inflammation as a risk factor improved the prediction of stroke (Ridker et al., 2000). Hs-CRP was the strongest univariate predictor out of the 12 markers measured. This is in relation to poor ANS activity. This relationship is tight enough to be considered as a reflex, the inflammatory reflex (Tracey, 2002). A confirmative cohort meta-analysis showed a linear association between CRP level and stroke (*P* = 0.940), as well as with CVD (*P* = 0.429), and CHD (*P* = 0.931); for each 1-mg/L increase in CRP level, the pooled RRs for stroke, CVD and CHD were 1.07, 1.18, and 1.12, respectively (Yang et al., 2021). Other studies found even higher risk predictive values, with a RR being 1.43 for CVD mortality (95% CI, 1.22–1.68) in the group with high CRP values (Ni et al., 2020). The strong relationship between ANS activity and inflammation allows to link these CVD risks to a defect in parasympathetic activity (Tracey, 2002; Doux and Yun, 2006; Ni et al., 2020; Rupprecht et al., 2020).

Another link for ANS implication in stroke occurrence is the independent association between IL-6 levels and cardiovascular events (Miwa et al., 2013). The chronic cardiovascular inflammation related to atherosclerosis-driven cerebrovascular disease is maintained through a failure in the resolution of inflammation (Bäck et al., 2019). Murine models of a disrupted parasympathetic signaling through vagotomy have demonstrated a reduction in specialized proresolving lipid mediators (Mirakaj et al., 2014), which exert key atheroprotecive effects (Arnardottir et al., 2021). Transcutaneous vagal neurostimulation (tVNS) protects from the increase in inflammation induced by administered lipopolysaccharide in rats (Zhao et al., 2012). However, if tVNS protects against inflammation in animal models, the long term effects have not been set in patients (Tynan et al., 2022).

Microglia activation due to sympathetic activation (Li et al., 2020) has severe consequences as inflammation favors neurodegeneration following stroke (Stuckey et al., 2021). Thus the ANS imbalance not only favors stroke occurrence but may also aggravate the post-stroke neurodegeneration.

### Autonomic nervous system as a predictive factor of stroke occurrence in the context of atrial fibrillation

Atrial fibrillation is becoming very frequent with an incidence reaching 1.01 and 2.16 for 100 person-years for the age ranges 65–74 and 75–84, respectively (Charlemagne et al., 2011). The incidence value may triple in 2050 (Miyasaka et al., 2006). Incidence is better measured through automatic long duration recordings than using patient-triggered devices (Roche et al., 1997, 2002). It was described as high as 83% after 85 years of age (Rajala et al., 1984).

Atrial fibrillation is an important predictor of stroke (Wolf et al., 1978, 1991; Harrison and Marshall, 1984; Flegel et al., 1987; Yamanouchi et al., 1989; Cabin et al., 1990; Broderick et al., 1992; Jerntorp and Berglund, 1992; Aronow et al., 1996a,b; Jorgensen et al., 1997). In the Framingham study, the risk ratio was 5.6, but reached 6.9 in the Whitehall Study of London Civil Servants cohort (Flegel et al., 1987). Even when not observed at the time of the stroke, AF is often suspected (Liao et al., 2007). Furthermore, silent strokes are frequently associated with AF (Yamanouchi et al., 1990, 1997a,b; Shinkawa et al., 1995; Barthelemy et al., 2003), the risk ratio reaching 2.5 (Yamanouchi et al., 1997b). The recent StrokeStop (Svennberg et al., 2021) and the Loop (Svendsen et al., 2021) studies were aimed at searching for AF and analyze the benefits of a preventive treatment in a monitored group versus a control group in terms of stroke incidence. They differ as the StrokeStop study recorded the ECG from intermittent patient intervention while the Loop study used an implanted loop recorder (ILR) (Svennberg et al., 2015). The StrokeStop Study determined a significant decrease in ischemic stroke (HR 0.76), while the Loop study did not, the incidence using IRL being equivalent to its control group. The differential results of the studies may reflect (a) the healthy user bias, (b) detection mode and burden of AF, (c) background detection, and (d) choice of endpoints (Svennberg and Braunschweig, 2021). Since hand-held single-lead ambulatory ECG screening twice daily for 2 weeks was able to detect AF for stroke-prevention, heart rate monitoring appears feasible with possible extension to a refined stroke prediction based on ANS activity.

Autonomic nervous system imbalance plays an important role in the initiation and maintenance of AF (Chen et al., 2014; Acampa et al., 2018), which is itself frequently associated with stroke. AF is frequently present in unexplained stroke (Barthelemy et al., 2003). AF accounts for uneven atrial conduction which can be induced by uneven sympathetic or parasympathetic stimulations of the atria. In relation to aging, the excess of sympathetic becomes easily predominant. The effects of NE, and nerve growth factor (NGF) on AF vulnerability have a relationship with the ionic remodeling, while the sympathetic hyperinnervation did not have a strong association with the induction of AF (Yang et al., 2019).

Heterogenous effective refractory periods (ERPs) are the hallmark of electrophysiological remodeling in AF. Although high parasympathetic tone prolongs ventricular ERPs, atrial ERPs are shortened and, importantly, this parasympathetic shortening is not uniform through the atria, rather, it is heterogeneously distributed even in healthy hearts (Linz et al., 2019). Adrenergic stimulation exerts most of its arrhythmogenic influence through increases intracellular Ca^++^. When this Ca^++^ loading is associated to an impaired CA^++^ reuptake mechanism, it permits favorable conditions for Ca^++^ triggered arrhythmic activity by inducing heterogeneously shortened action potential duration (Pfenniger et al., 2021).

Because of its pharmacologic property of preferentially blocking the N-type calcium channel, the dual (L-/N-) calcium channel blocker cilnidipine primarily blocks release of norepinephrine from sympathetic nerves, with a 20-fold smaller effect on L-type calcium channels. Evaluation in the canine atrial tachypacing model of AF showed that this compound could attenuate norepinephrine release and reduce electrical remodeling (both ERP and conduction velocity) as well as structural remodeling (Tajiri et al., 2019).

On the acetylcholine side, high sequence homology between all isoforms of muscarinic receptors (M1–M5) makes difficult the development of highly selective M2 inhibitors (Aistrup et al., 2009).

Methods that reduce autonomic innervation or outflow have been shown to reduce the incidence of spontaneous or induced atrial arrhythmias, suggesting that neuromodulation may be helpful in controlling AF (Chen et al., 2014).

Furthermore, the autonomic atrial receptors can themselves be unevenly stimulated as a consequence of autoantibodies. At least three types of autoantibodies have been found: anti-myosin, anti-M2 muscarinic receptor, and anti-heat shock protein autoantibodies. The best evidence concerns the M2-autoantibody. The proarrhythmic effect of the patients’ purified immunoglobulin G containing anti-M2 autoantibodies was confirmed by the occurrence of atrial premature contraction using IgG from patients with AF either idiopathic or in relation with dilated cardiomyopathy (Baba and Fu, 2008).

Any cause of parasympathetic decreased activity gives its full place to adrenergic activity, which is a strong risk enhancer of AF. An atrial parasympathetic receptor decrease due to autoantibodies eventually leads to AF (Baba et al., 2004; Zou et al., 2013; Gurses et al., 2015). This is also confirmed by prediction of AF recurrence when these antibodies are present in post pulmonary vein isolation (Gurses et al., 2015). In such conditions, strong sympathetic hyperactivity seems of particular frequency and importance (Linz et al., 2014).

The lack of parasympathetic activity is by itself a major factor of unbalance, since vagal neurostimulation (VNS) either acute or progressive is able to resolve AF (Kulkarni et al., 2021). Indeed, experimental studies and clinical trials that have explored the effects of neurostimulation on cardiac autonomic control have related to HF, ventricular arrhythmia and AF.

An intensity-dependent effect of vagal activation has been demonstrated in AF (Coumel et al., 1978; Bettoni and Zimmermann, 2002; Patterson et al., 2005). Thus high intensity vagal stimulation, i.e., at a level sufficient to slow sinus rhythm or atrioventricular conduction rate, induced AF. On the other hand, low intensity stimulation, below the threshold for slowing down the activity of the nodal tissue, exerted an anti-arrhythmic influence (Li et al., 2009; Shen et al., 2011; Sheng et al., 2011; Yu et al., 2011).

In anesthetized dogs, chronic low intensity stimulation of the tragus has been shown to protect against the development of AF induced by direct mechanical auricular stimulation (Li et al., 2009). In general, a shift in the sympathovagal balance in favor of the parasympathetic is observed among patients responding favorably to tVNS (Popov et al., 2013). Also, in people referred for AF ablation, Stavrakis et al. (2015) demonstrated an antiarrhythmic and anti-inflammatory effect of tVNS. In this study, patients under anesthesia received an auricular stimulation to induce AF. With stimulation, the duration of AF was significantly reduced as well as the blood concentration of inflammatory markers TNFα and CRP (Stavrakis et al., 2015).

Stavrakis et al., 2015, 2017, 2020 previous studies supported that tVNS could greatly facilitate induction and maintenance/resolution of AF according to the intensity of the stimulation. Indeed, (i) a strong vagal stimulation leads to an important sinus rate slowing, which promotes AF inducibility, whereas (ii) a low intensity stimulation produces antiarrhythmic effects. Moreover, Stavrakis et al. (2017) showed that electrical stimulation of the vagus nerve at levels substantially below the bradycardia threshold decreased the incidence of postoperative AF (POAF) and suppressed inflammation induced by cardiac surgery.

These observations suggest that vagal stimulation can exert either proarrhythmic or antiarrhythmic effects on the atrial function based on the intensity of stimulation.

### Autonomic nervous system as a predictive factor for stroke occurrence in the context of hypertension and blood pressure variability

Hypertension is a clinical landmark of stroke occurrence (Beckett et al., 2008). The relationship is continuous, and independent of other risk factors. The risk for death from and stroke increases steadily beginning at SBP as low as 115 mm Hg. The mortality of stroke double with each increment of 20 mm Hg SBP (Chobanian et al., 2003; Grysiewicz et al., 2008). The Hemorrhagic Stroke Project reported an adjusted odds ratio of 5.71 for hypertension among hemorrhagic stroke cases compared with age-matched controls (Feldmann et al., 2005). There is a tight relationship between hypertension and excessive sympathetic activity (Mancia and Grassi, 2014). Also, hypertension accelerates vascular ischemic disease, as does sympathetic activity (Mancia and Grassi, 2014). There is also an increase in the density of β-adrenergic receptors (Brodde et al., 1984).

Autonomic regulation of BP is of major importance. Autonomic regulation of blood pressure involves several factors, including heart rate, myocardial contractility, and vascular resistance. Baroreflexes are the major factor of BP modulation (Mancia et al., 1986). This system is rather simple, reporting information relative to distension of carotid and aortic arch and inducing a decrease in BP. The information sent to a central regulator located in the brainstem is updated at each arterial pulse. The neuronal output drives a parasympathetic command which lowers BP and slows heart rate. Unfortunately, this reflex, the baroreflex, which depends on a mechanical sensitivity to artery stretching, decreases with age (Abboud, 2010). The consequences of baroreflex impairment are chronic increased levels of BP, an impaired ability to respond to acute challenges to BP stability, low blood pressure periods, and an associated increased risk of sudden cardiac death.

The combination of CKD and hypertension is a situation in which an excess of sympathetic activity is very deleterious. Renal sympathectomy has demonstrated some advantages.

BP regulation is highly dependent on the ANS. In spontaneously hypertensive rats, there was a relationship in BP with the age-related loss of cardiac vagal preganglionic neurons (Corbett et al., 2007). Baroreflex impairment with age in humans also depends on loss of vagal innervation (Sagawa, 1983; Monahan, 2007). In the subjects aged 65 years old from the PROOF cohort, there are strong correlations between ANS dysregulation and hypertension (Dauphinot et al., 2013). On short term studies, Non-Esterified Fatty Acids (NEFA) can raise blood pressure, heart rate, and α1-adrenoceptor vasoreactivity, while reducing baroreflex sensitivity, endothelium-dependent vasodilatation, and vascular compliance (Egan, 2003).

The control of hypertension protects against stroke at any age (Bjorklund et al., 2004). An intensive approach targeting a BP below 140 mm Hg determined a hazard ratio of 0.75 of fatal and non-fatal cardiovascular events in the general population (Group et al., 2015).

Blood pressure variability adds also a predictive power to CV events. Methods to measure BPV measurements vary from repeated intra-daily measurements, successive daily or monthly measurements as well, the most common representation being standard deviation of 24-h ambulatory blood pressure (ABP) measurements. Other approaches propose to calculate the coefficient of variation of successive measurements, which is their SD divided by mean blood pressure, as well as many other parameters (Andalib et al., 2020). Indeed, current ambulatory recording devices do not give individuals successive systolic, or diastolic, peaks, and the calculations performed on ambulatory BP measurements are thus performed on already averaged data.

Blood pressure variability is a significant predictor of CV events including stroke as shown from the Uppsala Longitudinal Study of Adult Men (ULSAM) where BPV, reflected by the standard deviation (SD) of daytime and night-time SBP, significantly predicted stroke (Bjorklund et al., 2004). Twenty-four hour ambulatory pulse pressure (PP) gave a HR reaching 1.29 for one SD increase in daytime ambulatory PP independently of other established CV risk factors (Bjorklund et al., 2004). In the Chinese residents in Pu-Li town and Kinmen county, Taiwan, average real variability (ARV) index recorded on a short period added significant prognostic information (Hsu et al., 2016). BPV is also associated with cerebral white matter hyperintensity and might be one of the pathophysiological phenomena involving in the small vessel disease independent of hypertension (Zhang et al., 2022). The Hisayama study showed that increased day-to-day BPV is, independently of average home blood pressure, a significant risk factor for the development of all-cause dementia (Oishi et al., 2017). BPV predicts also CV events after a first stroke (Dawson et al., 2000; Kakaletsis et al., 2022). Regardless of whether they had hypertension, higher visit-to-visit SBP variability was significantly associated with a higher risk of MACE (Liu et al., 2022). Nighttime BPV was said to contribute to the prediction of CV events (Palatini et al., 2014) while this may be related to OSA. Also, the BPV prediction analysis showed that, in a large population cohort, which provided sufficient statistical power, BPV assessed from 24-h ambulatory recordings did not contribute much to risk stratification over and beyond 24-h BP (Hansen et al., 2010).

Due to its central role in blood pressure regulation, the baroreflex dysfunction may increase BP as well as disrupts BPV, and become a significant marker (Kaufmann et al., 2020).

### Autonomic nervous system as a predictive factor for stroke occurrence in the context of vascular stiffness

Arterial stiffness is a recognized risk factor for stroke and patients with acute ischemic stroke show higher arterial stiffness index values (Tuttolomondo et al., 2010). Among stroke patients, lacunar subtype has the highest arterial stiffness indexes, underlining the relationship between vascular aging and endothelial dysfunction (Tuttolomondo et al., 2010, 2017). Abboud underlined the relationship between endothelium health and ANS activity (Abboud, 2010). The combination with OSA is also underlined by the association with arterial stiffness (Saeed et al., 2022). Arterial stiffness is also an independent risk factor for hemorrhagic transformation in ischemic stroke undergoing thrombolysis (Acampa et al., 2017). Increased arterial stiffness is consistently associated with the presence of deep cerebral microbleeds and severe enlarged perivascular spaces burden at the BG and CS (Bae et al., 2021). Direct evidence of the dependence of artery stiffness from sympathetic activity was demonstrated (Holwerda et al., 2019; Tsioufis and Dimitriadis, 2019).

### Autonomic nervous system as a predictive factor for stroke occurrence in the context of sleep apnea

The prevalence of sleep apnea varies among studies. In the Wisconsin Sleep Cohort (WSC) study, the prevalence was as high as 24% for men and 9% for women. In Europe, the prevalence is around 10% (Heinzer et al., 2015; Haba-Rubio et al., 2016). In the PROOF cohort, it was set at 56% in people aged 67 years (Assoumou et al., 2012a,b). The Hypnolaus study from Lausanne showed a prevalence reaching 23.4% in women and 49.7% in men, with a median age of 57 years (range 40–85) (Heinzer et al., 2015).

The profile of autonomic function found in OSA includes increased sympathetic activity and reduced parasympathetic activity (Sequeira et al., 2019). Overactivation of the sympathetic nervous system was shown to be positively correlated with hypoxia, negative intrathoracic pressure swings, and recurring cortical arousals (Ucak et al., 2021). In patients suffering from OSA, the arousal index strongly correlated with LF/HF ratio and VLF, markers of ANS imbalance (Sforza et al., 2007), they were also negatively correlated with the vagally mediated HF components (Ucak et al., 2021). The PROOF study shows strong correlations between ANS dysregulation and OSA (Roche et al., 2003, 2009, 2012; Barthelemy et al., 2007). Furthermore, autonomic sympathetic activation during sleep predicts new-onset ambulatory hypertension (Roche et al., 2012). These strong relationships reinforce the interest in using Holter systems to detect OSA from Holter ECG (Pichot et al., 2015; Lao et al., 2021), and ANS activity may also be particularly well suited to follow treatment efficacy (Kim et al., 2020).

Altered autonomic function in OSA was implicated in increased cardiovascular risk (Dissanayake et al., 2021). Autonomic abnormalities in OSA are associated with a 46% greater risk of cardiovascular events (Fang et al., 2020). These vascular risks were well described, but the authors did not discriminate cardiact events from cerebral events (Marin et al., 2005). The development of cardiovascular disease in OSA is multifactorial and induces a cascade of events. The primary contributing factor is sympathetic overactivity (Ucak et al., 2021). Even unrecognized sleep apnea is associated with elevated ABP (Roche et al., 2012), elevated CRP values (Roche et al., 2009), and cardiac arrhythmias (Roche et al., 2003).

### Autonomic nervous system as a predictive factor of stroke occurrence in the context of combined factors

Interactions are most frequently linked to sympathetic hyperactivity brought on by acute and mostly chronic stress. Cerebrovascular risk factors are often associated.

A less clinically patent risk factors is probably sleep apneas are there is no biological markers and no obvious clinical marker as obesity or hypertension while sleep apneas may be at the origin of several established cerebrovascular risk factors (Van Ryswyk et al., 2018).

Sleep apnea accounts for a chronic intermittent stress and strongly leads to increased AF (Braga et al., 2009; Tavares et al., 2021), hypertension (Peppard et al., 2000), and dyslipidemia. Sleep apnea, obesity, diabetes mellitus, hypertension, and more generally the periods before AF, all share a decrease in ANS activity as a common factor of stroke occurrence. Interestingly, ANS activity, specifically the parasympathetic activity, may be enhanced through exercise training which may benefits sleep apnea disorders (Berger et al., 2018, 2019). The chemoreceptor reflex contribution to the pressor response is usually small compared with the baroreflex contribution, but it becomes very significant and predominant with senescence (Abboud, 2010). Furthermore, baroreflex impairment is associated with an enhanced chemoreceptor sensitivity resulting in a major sympathetic stimulation (Abboud, 2010). In patients with cardiac heart failure (CHF), untreated OSA is associated with an increased risk of death independently of confounding factors (Wang et al., 2007). Chronic artificial baroreceptor activation enhances survival in dogs presenting a pacing induced heart failure (Zucker et al., 2007). This stimulation suppresses the increases in plasma norepinephrine (NE) and angiotensin (ANG II).

Controlling one of combined factors can be beneficial, as refers to diabetes, where the control of hypertension divides the occurrence of stroke by a factor three (Bragg et al., 2021).

The PROOF cohort study demonstrated frequent arousals from sleep, with or without hypoxia, to increase BP and put healthy elderly volunteers at an higher risk of hypertension and thus of stroke (Chouchou et al., 2014). The importance of not interrupting sleep cycles, to allow to reach profound sleep states, has been illustrated (Jurysta et al., 2003). Any sleep interruption prevents the sleep deepening and will thus not permit parasympathetic activation. Hypoxia determines a strong supplementary sympathetic stimulation through chemoreceptors activation. This stimulation further decreases the parasympathetic activity through a central counter action, multiplying further the aggressivity of adrenergic hormones.

One risk factor can easily be associated with many others. Interestingly, the PROOF cohort study pointed out a linear relationship between a decreased baroreflex, which is a major factor of parasympathetic activity, and the number of components of metabolic syndrome (Assoumou et al., 2012a).

Even the memory performance was shown to be dependent on ANS. This underlines the need for a permanent high level of parasympathetic activity for a preserved long-term brain microvascular health (Saint Martin et al., 2015).

## Some views on a common consequence of autonomic nervous system unbalance: Neuroendothelial disease

Endothelial function is highly dependent on ANS activity which regulates blood flow as well as tissue-blood exchanges. During severe biological unbalance, as hypoxia or acidosis, the endothelium function may be severely compromised. Inflammation is a factor common to many pathologies and dysfunctions. Endothelial dysfunction may be found in each of the chronic diseases just described.

Endothelium disease is associated to AF (Corban et al., 2021). The decrease in parasympathetic activity associated with an increase in sympathetic activity plays a key role in vascular vulnerability (Bäck et al., 2019). This triggers several pathways including increased reactive oxygen species (ROS) and increased inflammation (Harada et al., 2015; Bäck et al., 2019). Beyond their high cellular aggressivity and disruptive effects on regulations, ROS further enhance sympathetic activity by stimulating the hypothalamic subfornical centers (Abboud, 2010). By this way, inflammation triggers a large increase in vascular inflammasome (Bäck et al., 2019). The disease further extends to the content of the vessels by activating platelets and many other factors (Harada et al., 2015).

Increased ROS induce microvascular dysfunction including impaired endothelium-dependent vasodilator and enhanced endothelium-dependent vasoconstrictor responses, along with increased vulnerability to thrombus formation (Yu et al., 2019). Increased ROS thus result in enhanced fluid filtration associated with protein extravasation and activation of leukocytes. All of these pathologic microvascular events involve the increased production of ROS, and consecutive induced ischemia reperfusion, which further activates inflammasomes, provokes severe mitochondrial disorders, and determines release of microvesicles in endothelial cells (Bäck et al., 2019). There is a relationship between HRV and endothelial function evaluated through brachial artery flow mediated dilation (Pinter et al., 2012). This approach underlines the strong dependency of endothelium to ANS parasympathetic activity. Chronic baroreceptor stimulation improves endothelium function (Chapleau et al., 2016), as does direct acetylcholine administration (Borovikova et al., 2000). Endothelium health is thus strongly dependent on parasympathetic activity. Rebalancing autonomic activity through VNS attenuates this dysfunction and protects from stroke (Carandina et al., 2021).

## The effects of autonomic nervous system improvement by neural stimulation

Both VNS and renal denervation are intended to increase parasympathetic activity and decrease sympathetic predominance. Acute, or more often chronic, VNS enhances endothelium health which enhances vagal protective activity (Saeed et al., 2005; Chapleau et al., 2016).

The powerful downregulation of inflammatory process by vagal stimulation is a fundamental key of the protection brought by vagal nerve activity. Transcutaneous auricular vagus nerve stimulation (tVNS) was shown to protect rats from the potent lipopolysaccharide inflammatory stimuli, by decreasing TNFα, IL-6, and IL-1β response to the inflammatory stimuli. Conversely, inhibition of the tVNS effects by α7nAChR antagonist injection confirmed this mechanism (Zhao et al., 2012). Using the Shwartzman reaction, nicotine and the CAP55 cholinergic agent decreased substantially both VCAM-1 mRNA and *E*-selectin mRNA expression by the endothelium, and reduce leukocyte adhesion (Saeed et al., 2005). *In vivo* VNS in the carrageenan air pouch model induced similar effects (Saeed et al., 2005). Vagal stimulation determines a peripheral vascular protection in a rat model of myocardial ischemia/reperfusion through the cholinergic anti-inflammatory pathway which is dependent on a7nAChR (Zhao et al., 2013).

In CHF, a strong negative correlation was shown between the plasmatic increase in NE and survival (Cohn et al., 1984). This underlines the central role of NE as a highly toxic endocrine factor. On the other hand, the strong protective effect of parasympathetic activity was demonstrated in CHF through vagal stimulation (Zucker et al., 2007). Neurovagal stimulation was shown to correct or help reducing AF (Kiuchi et al., 2016; Stavrakis et al., 2020). Low-intensity vagal stimulation inhibits AF in an animal model of OSA (Gao et al., 2015). In another critical clinical field, CKD, complete denervation of the native diseased kidney by bilateral nephrectomy decreases the cardiovascular risk (Obremska et al., 2016).

After an induced ischemic stroke, infarct size was significantly reduced in response to vagal stimulation in animals (Yang et al., 2018); the infarct size decrease was associated with a decrease in blood-brain transfer in the stimulated group, spatially correlated with the attenuation of the infarct size. It was also shown that vascular tight junctions were protected in microvessels with lower serum proteins leakage, which underlines the protection brought to the endothelium. After stroke, the non-invasive VNS reduces blood-brain barrier disruption in a rat model of ischemic stroke (Yang et al., 2018).

## Conclusion

Today, since Hilz et al. (2011) proposed to substitute the NIH Stroke Scale (NIHSS) clinical values score to encompass the lack of ANS variables availability and take advantage of that ANS variables have become readily available through accessible devices and software (Pichot et al., 2016). If either low ANS variables values or ANS severe imbalance is detected, a priority should be to identify the disorder explaining the fall in ANS activity and taking the needed corrective decisions to avoid further disease extension. This includes mainly OSA, hypertension, diabetes, CKD, and inflammation. Recording ANS activity in large populations should be encouraged to measure ANS on large populations, the recordings may be validated through cardiologist teams to ensure the needed quality.

Vagal activity is a key factor which, when maintaining its activity through physical exercise is not possible, may be boosted by non-invasive transcutaneous vagal stimulation.

Obtaining individual values only needs analysis of an ECG recording, preferably on a full nyctohemeral period, which is now easy to perform and repeat. Using growth curves for sympathetic and parasympathetic activity would be straightforward using growth curves similar to children’s weight and height. An annual recording may be a good preventive target. The recording may be performed at young age when unfavorable clinical conditions are present.

The predictive power of ANS activity for cardiovascular diseases may lead to a large utilization of autonomic modulation in preventing the occurrence of ischemic and hemorrhagic stroke and limiting their severity (Carandina et al., 2021). We may well have the age of our autonomic nervous system and we can enhance its protective activity through physical exercise or, if is difficult to exercise, through tVNS.

## Author contributions

J-CB, VP, DH, MBe, SC, LM, MBä, J-RL, and FR contributed to conception and design of the study. J-CB wrote the first draft of the manuscript. All authors contributed to manuscript revision, read, and approved the submitted version.
